# Wernicke encephalopathy in a patient with drug-induced liver failure: a case report

**DOI:** 10.3389/fnut.2024.1505974

**Published:** 2025-01-07

**Authors:** Jiao-Jiao Cao, Jing Li, Yan Cheng, Li-Min Luo, Yang Chen, Chang-Xing Huang, Ye Zhang

**Affiliations:** ^1^Department of Infectious Diseases, Tangdu Hospital, Fourth Military Medical University, Xi'an, Shaanxi, China; ^2^Department of Infectious Diseases, Air Force Hospital of Southern Theatre Command, Guangzhou, Guangdong, China; ^3^School of Basic Medicine, Fourth Military Medical University, Xi'an, Shaanxi, China

**Keywords:** Wernicke encephalopathy, non-alcoholic, drug-induced liver injury, liver failure, vitamin B1

## Abstract

**Introduction:**

Wernicke encephalopathy is a metabolic disease mainly associated with vitamin B1 deficiency, which is common in chronic alcoholism. Non-alcoholic Wernicke encephalopathy is difficult for early diagnosis.

**Case presentation:**

One case involved a 62-year-old man who was admitted to hospital with drug-induced liver failure. He presented lower extremity weakness and progressive worsening of consciousness disturbance post-admission and was eventually identified as Wernicke encephalopathy by magnetic resonance imaging scan and deficiency in vitamin B1. The classic symmetric hyperintense signals on T2-weighted and diffusion-weighted images were reversible after intravenous vitamin B1 supplementation.

**Conclusion:**

A high index of clinical suspicion is required for early diagnosis and appropriate preventive and therapeutic strategies by adequate and immediate vitamin B1 supplements in the reversible stage of Wernicke encephalopathy.

## Introduction

Wernicke encephalopathy is a metabolic disease mainly associated with vitamin B1 deficiency which leads to permanent brain injury and life-threatening complications (1). Wernicke encephalopathy is common in chronic alcoholism, but non-alcoholic Wernicke encephalopathy is difficult for early diagnosis due to the various presentations. In this study, we reported a non-alcoholic patient with drug-induced liver failure who developed Wernicke encephalopathy.

## Case report

A 62-year-old man was admitted to our department for jaundice and new onset ascites for 2 days. He had a history of administrating herbal medicine for 6 months due to the diagnosis of lung nodules in a routine physical examination. He was noted to have progressive fatigue and poor appetite 2 weeks before the admission. Laboratory evaluation yielded the following: white blood cell count, 7.19 × 10^9^/L; neutrophil ratio, 70.4%; red blood cell count, 5.18 × 10^12^/L; hemoglobin, 158 g/L; platelet count, 178 × 10^9^/L; total bilirubin, 344.1 μmol/L; alanine aminotransferase, 830 U/L; aspartate aminotransferase, 966 U/L; alkaline phosphatase, 123 U/L, gamma-glutamyl transferase, 135 U/L; albumin, 30.6 g/L; blood ammonia, 35.20 μmol/L; blood urea nitrogen, 14.4 mmol/L; creatinine, 116.0 μmol/L; prothrombin activity, 41.6 %; international normalized ratio, 1.74; and alpha-fetoprotein, 21.80 ng/mL. The results were negative for hepatitis viruses, human immunodeficiency virus-1, cytomegalovirus, Epstein–Barr virus, parvovirus B19, and autoimmune diseases. Abdominal ultrasonography showed diffuse changes in the liver and ascites. He was diagnosed with drug-induced liver failure based on the medical history and the symptoms of acute hepatic insult.

**Figure 1:**
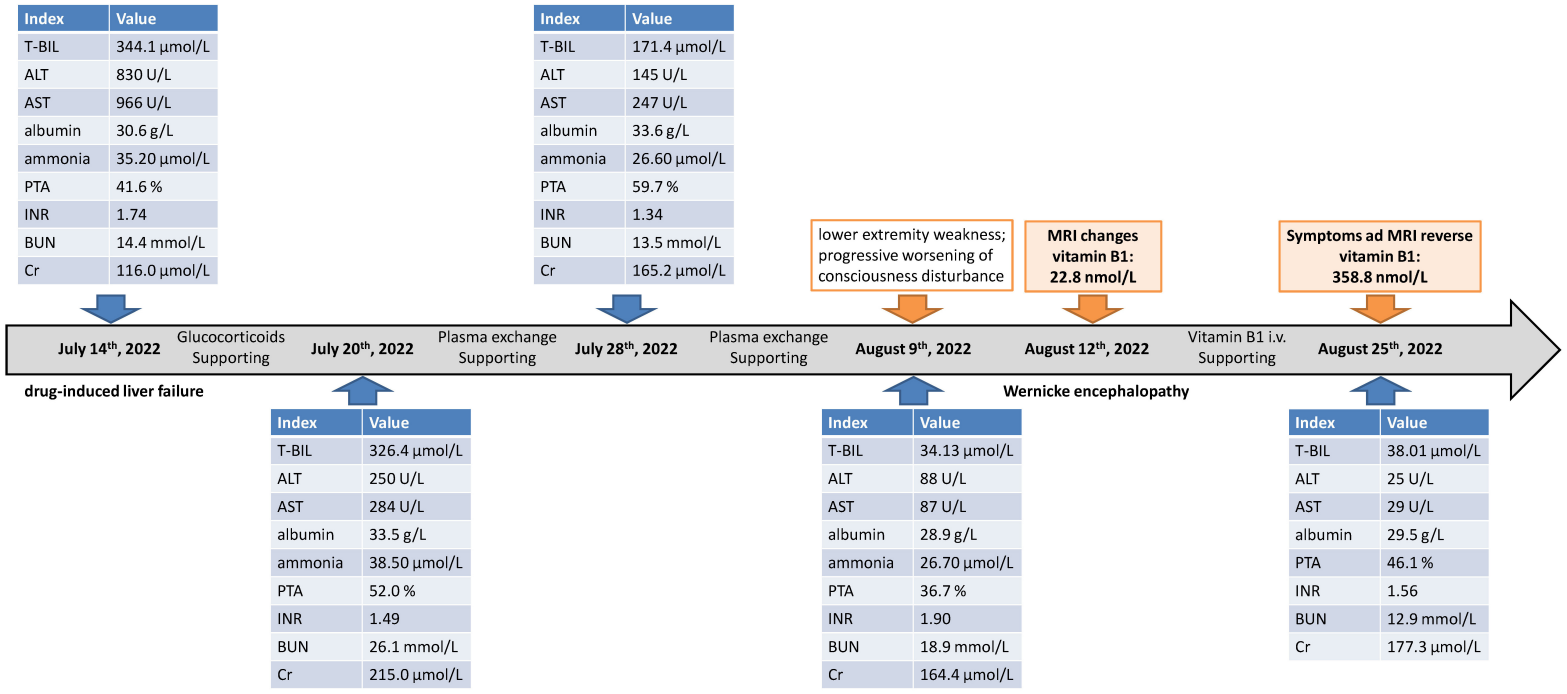
Showcase of timeline with the changes in liver function, coagulation function, renal function, and vitamin B1. T-Bil, total bilirubin; ALT, alanine aminotransferase; AST, aspartate aminotransferase; PTA, prothrombin activity; INR, international normalized ratio; BUN, blood urea nitrogen; Cr, creatinine; MRI, magnetic resonance imaging; i.v., intravenous.

**Figure 2:**
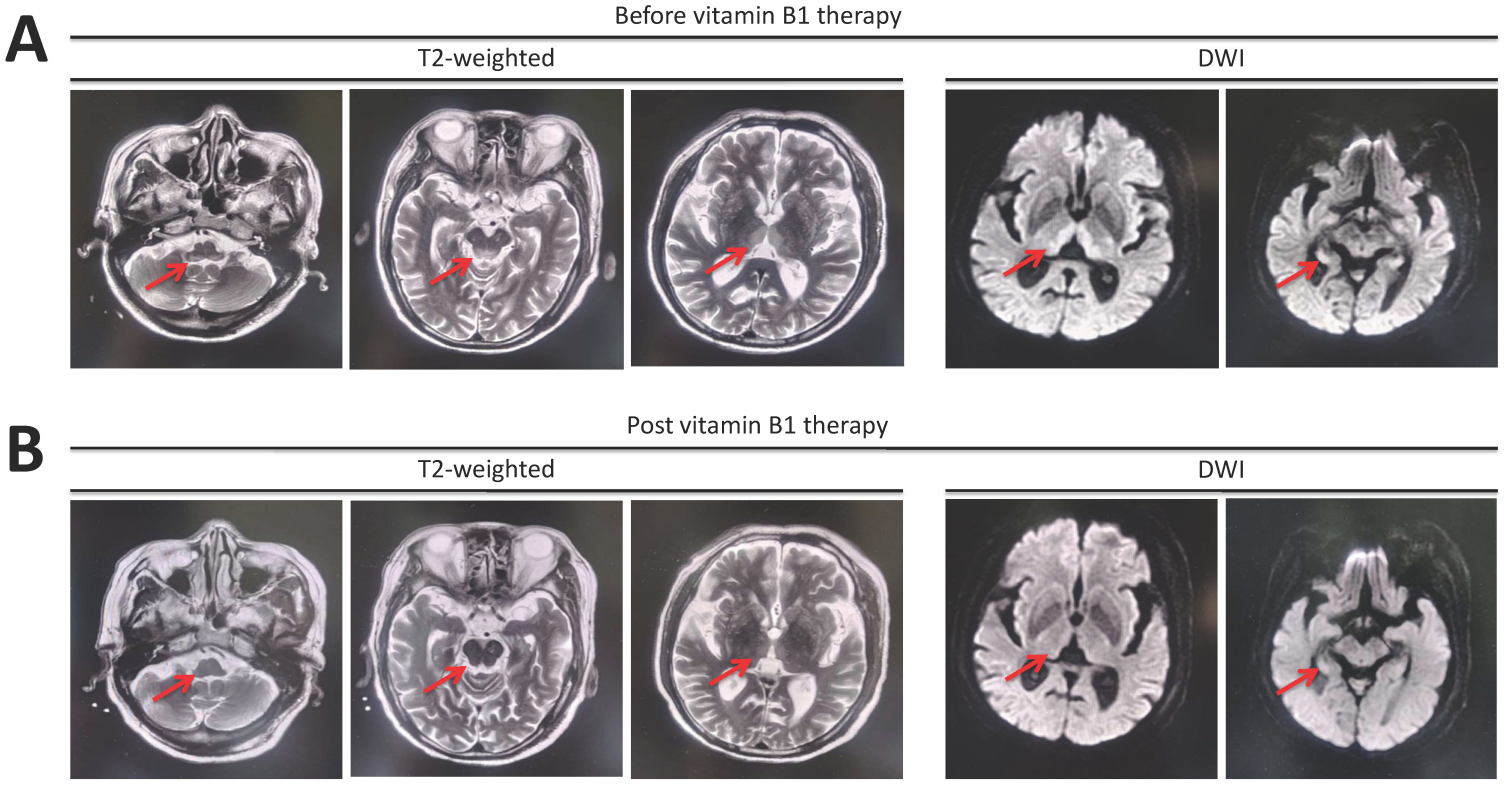
Magnetic resonance imaging manifestation reveals **(A)** symmetric hyperintense signals on T2-weighted images in the bilateral inferior cerebellar peduncle, dorsal pons, and medial thalami as well as increased signal intensities on diffusion-weighted images (DWI) in bilateral thalami and hypothalamus. **(B)** The lesions were reversible after 12 days of intravenous vitamin B1 supplementation.

He was treated with supportive measurements (including liver protective treatments, glucocorticoids, prophylactic antibiotics, diuresis, and lactulose) and plasma exchange. The laboratory parameters for liver and renal function were progressively improved (Figure 1). However, the patient had very poor appetite, and always nausea and vomiting after meals. Three weeks post-admission, he presented lower extremity weakness and progressive worsening of consciousness disturbance, manifesting as dysphoria, ecmnesia, and delirium to light coma. A neurological evaluation was then conducted. Thyroid function parameters, adrenal hormones, blood ammonia, blood sugar, and electrolyte levels were within normal limits. The result of the electrocardiographic examination was normal. The symptoms were not reserved with anti-hepatic encephalopathy therapies. Cerebral magnetic resonance imaging (MRI) showed symmetric hyperintense signals on T2-weighted images in the bilateral inferior cerebellar peduncle, dorsal pons, and medial thalami as well as increased signal intensities on diffusion-weighted images within bilateral thalami and hypothalamus (Figure 2A). He was vitamin B1-deficient at a level of 22.8 nmol/L (normal range: 70–180 nmol/L). He was diagnosed with Wernicke encephalopathy and immediately received intravenous vitamin B1 supplementation (200 mg per 8 h). His neurological symptoms improved, serum vitamin B1 level returned to 358.8 nmol/L (Figure 1), and the lesions in MRI were reversible 12 days post-vitamin B1 replacement therapy (Figure 2B). The vitamin B1 administration was changed to oral supplementation (200 mg/day) for 2 weeks. He was regularly followed up for 18 months after discharge, and no abnormalities were found during the follow-up period.

## Discussion

Wernicke encephalopathy was first reported in chronic alcoholism (2) and has occasionally been described as malnutrition due to a variety of causes, such as gastrointestinal surgery (3), organ transplantation (4), and upper gastrointestinal obstruction (5). Wernicke encephalopathy has also been reported in hepatitis B liver failure (6, 7). In this case, the patient did not have a history of chronic liver diseases or alcoholism but had a history of herbal medicine and was diagnosed with drug-induced liver failure. Poor appetite, insufficient dietary intake, and vomiting caused by impaired liver and renal function mainly contribute to the development of Wernicke encephalopathy. However, for patients with liver failure, the differential diagnosis of hepatic encephalopathy and Wernicke encephalopathy might be a tough problem in an emergency condition. In this case, the patient had normal blood ammonia and did not respond to anti-hepatic encephalopathy treatments. The symmetry variability in MRI revealed the metabolic encephalopathy, and vitamin B1 deficiency further confirmed the diagnosis of Wernicke encephalopathy.

Vitamin B1 can only be taken in from food and can neither be synthesized nor stored in the human body. Thiamine pyrophosphate is the biologically active form of vitamin B1 and plays a vital role in the tricarboxylic acid cycle. Vitamin B1 deficiency results in lactic acid accumulation and acidosis, thereby interfering with neurotransmitter production, release, and re-uptake, and finally leads to Wernicke encephalopathy (1). The degree of brain congestion and edema will be further aggravated in Wernicke encephalopathy due to the failure of prompt vitamin B1 supplementation (4). In this case, the usage of glucocorticoids might increase the consumption of vitamin B1, leading to the aggravation of Wernicke encephalopathy.

Three clinical components of Wernicke encephalopathy are impaired consciousness, ophthalmoplegia, and gait ataxia. However, the classical triad is only fully recognized in 10–38% of patients (8). The clinical diagnosis of Wernicke encephalopathy in alcoholics requires two of the following four signs, including (i) dietary deficiencies, (ii) eye signs, (iii) cerebellar dysfunction, and (iv) either an altered mental state or mild memory impairment based on the diagnostic criteria proposed by 2010 European Union of Neuroscience Association (8). Although the patient in this case was not an alcoholic, he still confirmed three of four elements. Furthermore, cerebral MRI is the most sensitive examination for the early diagnosis of Wernicke encephalopathy. The sensitivity and specificity of cerebral MRI for the diagnosis of Wernicke encephalopathy are 53% and 93%, respectively (9). Basal ganglia and thalamic region are mostly involved because these regions seem to be particularly vulnerable to oxygen deprivation (5) and presented symmetric high T1, T2, and T2 flair signal intensities (10). His cerebral MRI supported Wernicke encephalopathy, the laboratory test confirmed vitamin B1 deficiency, and he rapidly recovered after vitamin B1 supplementation without any sequelae. We definitively diagnosed Wernicke encephalopathy during drug-induced liver failure.

Once diagnosed or even suspected as Wernicke encephalopathy, the patient should immediately receive vitamin B1 administration, preferably intravenously with 200 mg thrice daily before any carbohydrate (8). The overall safety of vitamin B1 is good since vitamin B1 is water-soluble and can easily be excreted through the kidney (8). In this case, the patient received intravenous vitamin B1 immediately upon consideration of Wernicke encephalopathy. His clinical symptoms improved, and the lesions in the MRI were reversed 12 days later. He received oral supplementation of vitamin B1 for another 2 weeks. Vitamin B1 therapy was safe and effective.

In summary, we reported Wernicke encephalopathy developing in a patient with drug-induced liver failure. Patients with liver failure should be on the alert for starvation-induced Wernicke encephalopathy. A high index of clinical suspicion is required for early diagnosis and appropriate preventive and therapeutic strategies by adequate and immediate vitamin B1 supplements in the reversible stage of Wernicke encephalopathy. Furthermore, it is important for vitamin testing and supplements in patients with liver injury, especially for those who have insufficient dietary intake.

## Data Availability

The original contributions presented in the study are included in the article/supplementary material, further inquiries can be directed to the corresponding author.
